# A review of human dog‐bite injuries in Kitui South subcounty, Kenya (2017–2021)

**DOI:** 10.1002/vro2.72

**Published:** 2023-10-09

**Authors:** Peris Kung′u, David Brodbelt

**Affiliations:** ^1^ Kenya Veterinary Association Nairobi Kenya; ^2^ Department of Pathobiology and Population Science The Royal Veterinary College Hatfield Hertfordshire UK

## Abstract

**Introduction:**

Dog bites continue to be a serious public health issue due to their association with the transmission of rabies virus. In Kenya, there are no studies estimating dog‐bite incidence. Annual mortalities resulting from dog‐mediated rabies are estimated at 523 (95% confidence interval 134–1100). The main objective of this study was to assess major risk factors associated with dog bites in Kitui South subcounty, Kenya, between 2017 and 2021.

**Methods:**

We recruited 387 dog‐bite patients (cases) and 387 non‐bite patients (controls) for the case–control study from the Mutomo Mission Hospital and the Ikutha Level 4 Hospital records. Multivariable logistic regression analysis evaluated the association between risk factors and dog‐bite cases. In the final model, pairwise interactions among variables were evaluated. The model fit was evaluated using receiver operating characteristics and area under the curve.

**Results:**

The study found that the dog‐bite incidence was highest in Kanziko ward in Kitui South subcounty. Fifty‐one percent (108 bites) of dog‐bite victims were children under 15 years of age, with 53% (*N* = 68) being men and 36% (*N* = 77) being bitten on the limbs. Dog bites mostly (44%, *N* = 93) occurred between October and December (short rainy season). Age group and season were identified as the most significant variables for high dog‐bite incidence in Kitui South subcounty.

**Conclusions:**

Promotion of responsible dog ownership and reinforcement of dog control policies may prove more effective in reducing dog‐bite injuries in Kitui South subcounty.

## INTRODUCTION

Dog bites are an important public health challenge globally, affecting millions of individuals and leading to fatalities, especially in children.^1^ Dog bites cause both physical and psychological injuries in human beings, which include post‐traumatic stress disorder, nerve laceration, deformity, long‐term scarring, open fractures, osteomyelitis, bacterial infections, tendon laceration and tissue loss.^2^, ^3^, ^4^ Some of the bacteria transmitted during dog bites include Pasteurella, Salmonella, Brucella, Leptospira and Staphylococcus, while the viruses transmitted include lyssavirus and norovirus.^5^ Dogs are responsible for the transmission of zoonoses, particularly rabies virus, where they account for more than 99% of human rabies cases and approximately 22,000 people die annually of rabies in Africa alone.^6^, ^7^ Rabies is considered to be endemic in Kenya, with an estimated 523 human deaths (95% confidence interval [CI] 134–1100) annually; hence, dog bites are an important public health risk.^8^

Although there is a dearth of information on the global estimates of dog‐bite incidence, studies suggest that tens of millions suffer from dog‐bite injuries, causing deaths in an estimated 59,000 individuals annually.^9^ In the United States, approximately 4.5 million dog bites take place annually, while an estimated 1870 bites per 100,000 population occur annually in the UK.^10^, ^11^ There is limited evidence on the frequency and risk factors for dog bites in Africa.^12^ A scoping review conducted in 2021 recorded 543,714 animal bite victims, with most of the fatal cases being male children between 0 and 14 years of age.^7^

There are no reported studies in Kenya that estimate dog‐bite incidence due to the fragmented surveillance systems and limited resources. A retrospective study conducted between 2011 and 2016 estimated that for the eastern and western parts of Kenya, all human cases of animal bites were approximately 289 bites per 100,000 population annually.^13^ Furthermore, the study found that 72% (*N* = 5208) of the bite cases were inflicted by owned dogs, with male children under 15 years having a higher chance of experiencing an animal bite injury. Kitui county had an estimated 1266 animal bites, out of which 91% (*N* = 1160) were from dogs.^13^ Similarly, another study conducted between 2016 and 2018 in Homabay county estimated an annual animal bite of 1571 of 100,000 population.^14^ A more detailed assessment of the incidence and risk factors associated with dog‐bite injuries in Kenya is warranted. Understanding the incidence and factors associated with dog bites is essential for developing effective prevention and control strategies.

This study aimed to record the frequency of dog bites and major demographic and seasonal risk factors in Kitui South subcounty. The study hypothesised that demographic and seasonal factors (age, sex and season) were associated with an increased risk of human cases of dog bites in Kitui South subcounty.

## METHODS

### Study area

**FIGURE 1:**
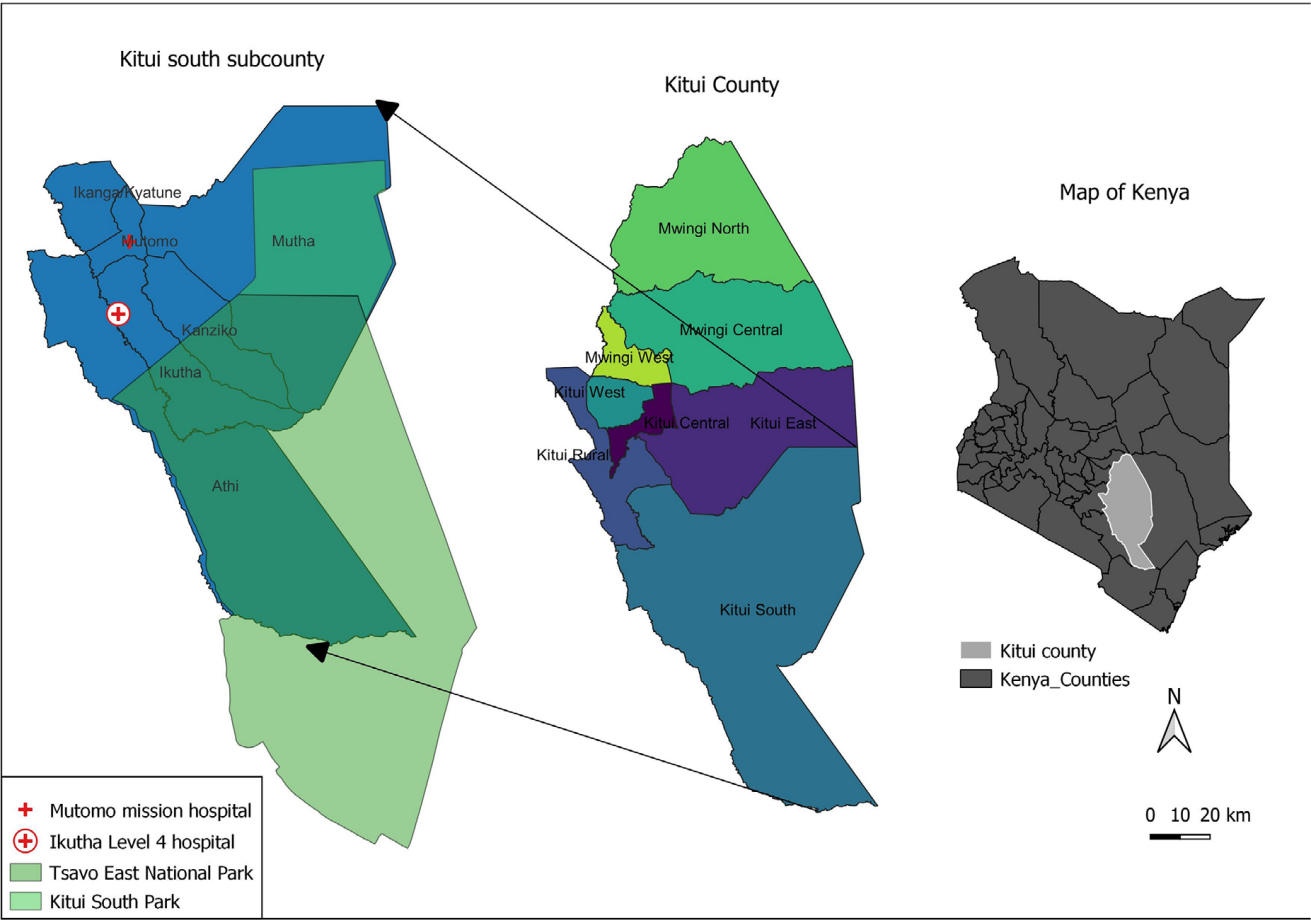
A map of Kitui county illustrating the locations of the two selected health facilities in Kitui South subcounty, Ikutha Level 4 Hospital and Mutomo Mission Hospital.

The study was conducted in Kitui South, a subcounty in Kitui county, which is situated in the southeastern part of Kenya. Kitui county has a human population of 1,136,187 and covers an area of 30,430 km^2^.^15^ The county consists of eight subcounties, namely, Mwingi North, Mwingi Central, Mwingi West, Kitui Central, Kitui East, Kitui West, Kitui Rural and Kitui South. Kitui South subcounty was purposefully selected as the study site due to its large human population (181,836) and ease of access to medical records. Kitui South subcounty has a total of 56 health facilities, of which two of hospitals were selected and agreed to participate (Ikutha Level 4 Hospital and Mutomo Mission Hospital [MMH]) in the case–control study (Figure 1).

### Study design and population

For the estimation of incidence of dog bites in Kitui South, a cohort study was undertaken based on the data extracted from the Kenya health information system (KHIS). The data included dog‐bite cases reported in Kitui South between 2017 and 2021. Dog‐bite cases were defined as an individual bitten by a dog and reported to a health facility. Descriptive and spatial analyses were then conducted. QGIS software (version 3.24.1; www.qgis.org/en/site/index.html) was utilised in the development of choropleth maps using calculated annual dog‐bite incidence rates from aggregate ward‐level dog‐bite injuries identified from the KHIS data (2017–2021) and the 2019 population Census data as the denominator. Dog‐bite incidence per ward was estimated with 95% CI. The dog‐bite incidence rates per year at the ward level were represented using five choropleth maps for the 5‐year study period. The annual dog‐bite incidence was also estimated at the hospital level for the two study hospitals (case–control dataset, see below) by using the number of dog‐bite cases as the numerator and the total number of patients visiting the hospital during the study period as the denominator.

The case–control study utilised data from patients attending two health facilities, which were designated as immunisation centres with access to anti‐rabies vaccines in Kitui South subcounty and hence likely to receive bite injuries. The study was conducted retrospectively in Ikutha Level 4 Hospital and MMH. The target population included patients attending MMH during the 5‐year period between 1 January 2017 and 31 December 2021 and due to limited access to data in the second hospital, those attending Ikutha Level 4 Hospital for 1 year between 1 October 2018 and 30 September 2019. Patient information at MMH was documented in an electronic hospital information management system, while at Ikutha Level 4 Hospital, it was documented via manually recorded outpatient records and an anti‐rabies vaccination register. The anti‐rabies vaccination register provided additional information on dog‐bite patient's demographics, injuries and their management.

In the case–control study, a case was defined as a person bitten by a dog and presenting to one of the two participating hospitals (MMH and Ikutha Hospital) in Kitui South subcounty. The variables collected from the cases included the date of hospital visit, patient demographics (age, sex), home geographical location of the patient (village, ward, subcounty), part of body bitten (leg, arm, feet, shoulder), dog ownership status (stray, owned) and post‐exposure rabies prophylaxis (PEP) (days 0, 3, 5, 14, 28). Controls were defined as individuals who presented at either MMH or Ikutha Hospital for a non‐bite reason during the respective study periods. Controls were randomly selected from the non‐bite patients at a 1:1 ratio to dog‐bite cases, stratified by hospital such that an equal number of controls was selected as cases within each hospital. The variables collected for controls included date of hospital visit, patient demographics (age, sex), home geographical location (village, ward, subcounty) and diagnosis or reason for hospital visit.

The sample size was estimated using the Open Epi software program.^16^ To identify a risk factor with an odds ratio of 1.5 or greater, based on a risk factor exposure in the controls of 50%, it was estimated that the sample size should include approximately 388 cases and 388 controls (1:1 case to control ratio, 80% power, 95% CI). A 1‐year period was elected for Ikutha Level 4 Hospital, where an estimated 47,500 patients attended the hospital between 1 October 2018 and 30 September 2019 due to availability of the data and the need to manually review the records. From MMH, the data were extracted from two hospital management systems utilised between 1 January 2017 and 31 December 2021. Initially, information was collected on all 71,868 patients who presented to MMH within the study period. Dog‐bite cases were identified from the electronic heath records. An equal number of controls was selected based on the number of cases at each hospital. The data collected were cleaned, checked for errors, saved to one Microsoft Excel sheet and then exported to R‐4.1.3 (R Core Team. R: a language and environment for statistical computing. Vienna, Austria: R Foundation for Statistical Computing; 2022. Available from: www.R‐project.org/) for descriptive, univariable and multivariable analysis.

For descriptive analysis, both categorical and numerical data were collected from the hospital records. Categorical data were summarised with number (percentage), while continuous data were evaluated graphically for their distribution and summarised through the mean (standard deviation) or median (range). In the case–control study, risk factors for dog bites were first explored in the univariable analysis with chi‐squared/Fisher's exact test for categorical variables and the Mann–Whitney *U*‐test for continuous variables, and crude odds ratios with 95% CIs were reported via univariable logistic regression. Variables broadly associated with bites (*p* < 0.25) were then taken forward for consideration in the multivariable logistic regression analysis. Independent variables taken forward for consideration in the multivariable analysis were evaluated for collinearity with the chi‐squared test. Where variables were highly related, only one of the related factors would be included in the model; for instance, season was included instead of month, while for age, the categorical variable was incorporated in place of the continuous age variable. The multivariable model was built in a manual forward selection construction approach and variables were retained if statistically significant. Pairwise interactions were assessed for the final model variables and the model fit was assessed using the area under the receiver operating characteristic curve. Statistical significance was set at the 5% level.

## RESULTS

### Incidence and distribution of dog bites in Kitui South, Kenya

**FIGURE 2:**
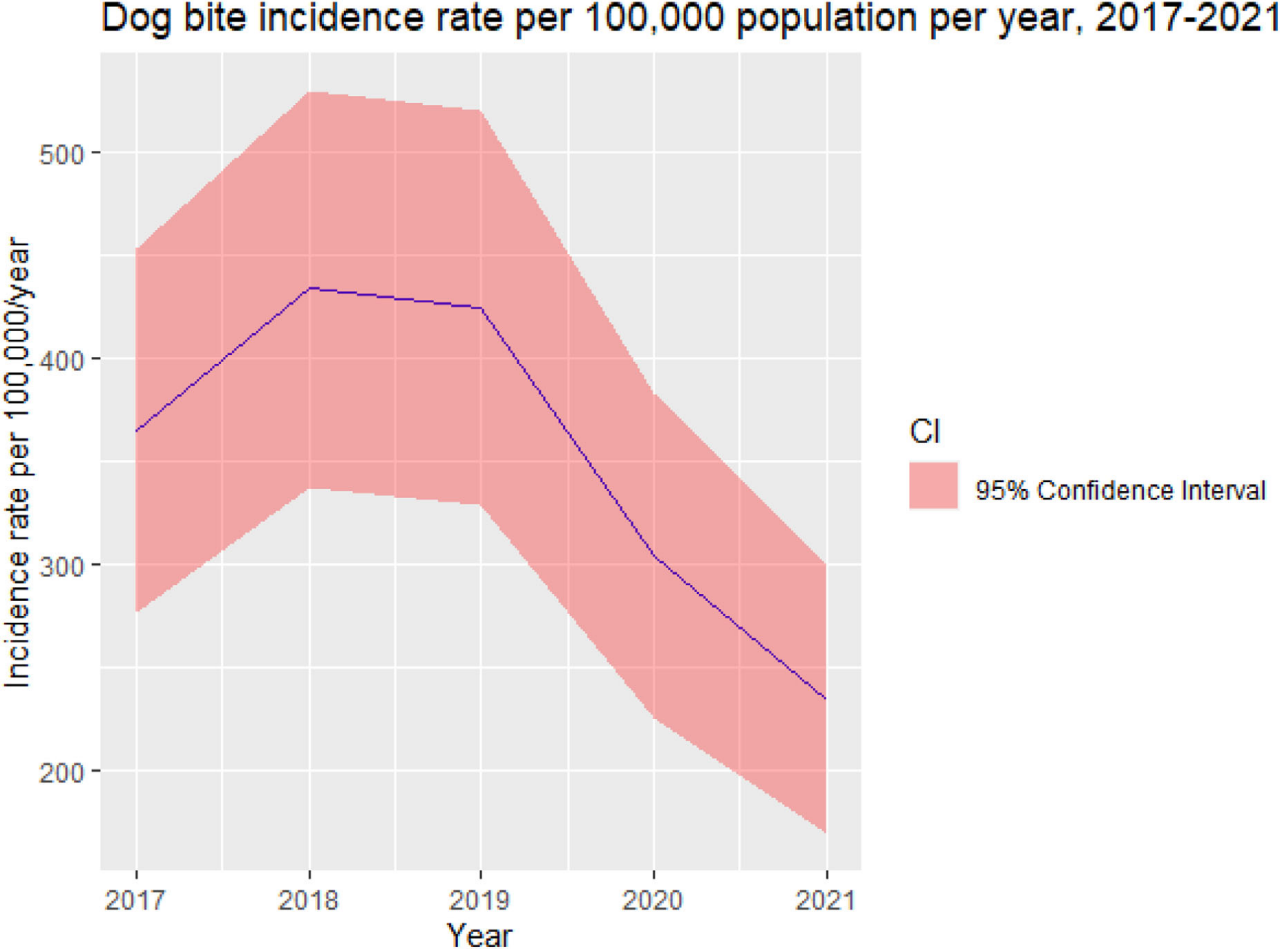
Graph illustrating dog‐bite incidence rate by year with confidence intervals (CIs) in Kitui South subcounty, Kenya, based on Kenya health information system data between 2017 and 2021.

A total of 3182 dog‐bite cases were obtained from the KHIS reporting system for Kitui South between 2017 and 2021. Figure 2 illustrates the trend of incidence risk by year, with the highest incidence rate of 433.6 (95% CI 395.1–474.7) per 100,000 population seen in 2018, which declined to 234.8 (95% CI 206.8–264.9) per 100,000 in 2021.

**FIGURE 3:**
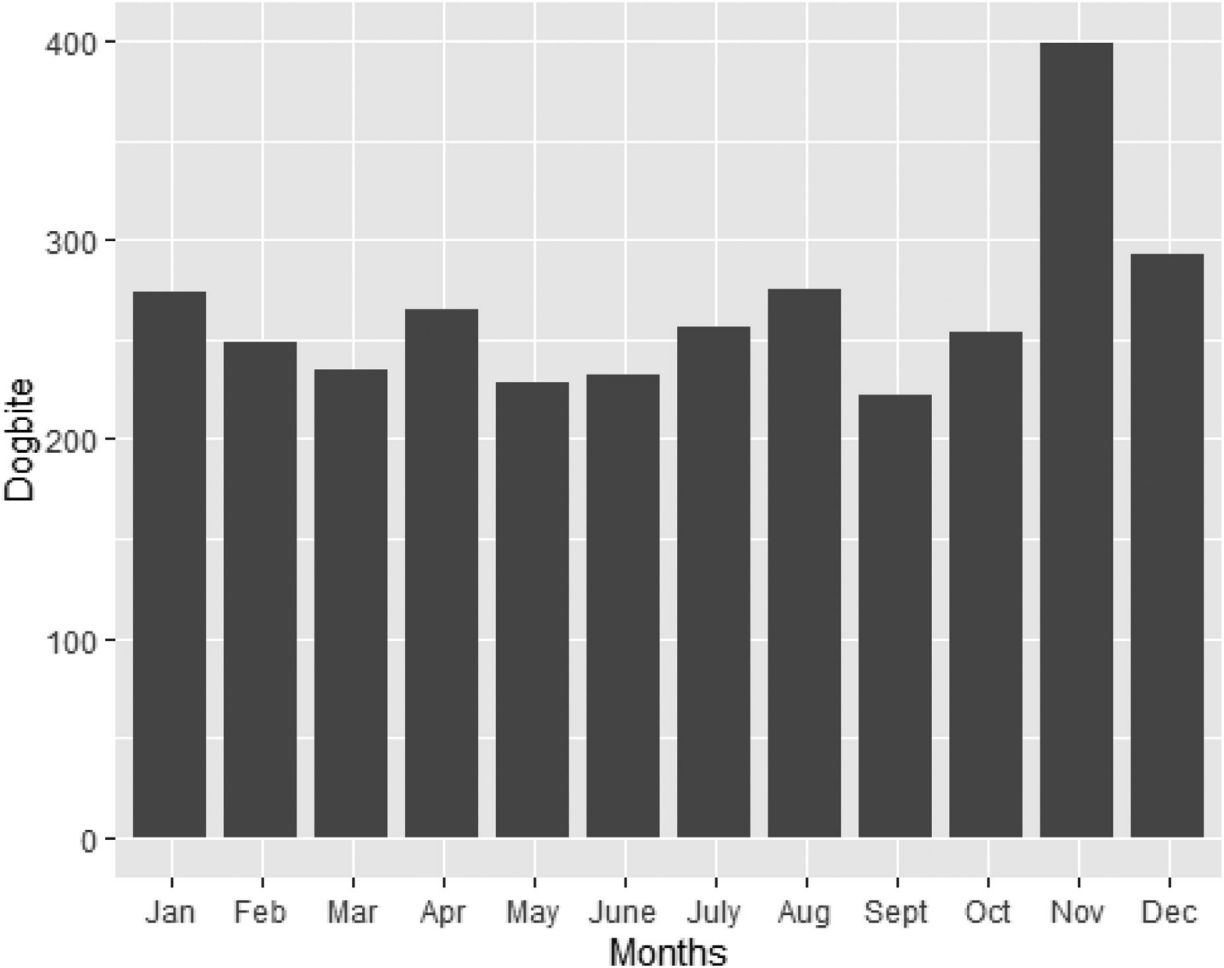
Bar graph showing the total dog‐bite injuries reported by month for the 5‐year period between 2017 and 2021 in Kitui South subcounty based on Kenya health information system data between 2017 and 2021.

In terms of the monthly dog‐bite distribution, slightly higher frequencies were observed in November (12.5%, *N* = 399) and December (9.2%, *N* = 293), as illustrated in Figure 3.

**FIGURE 4:**
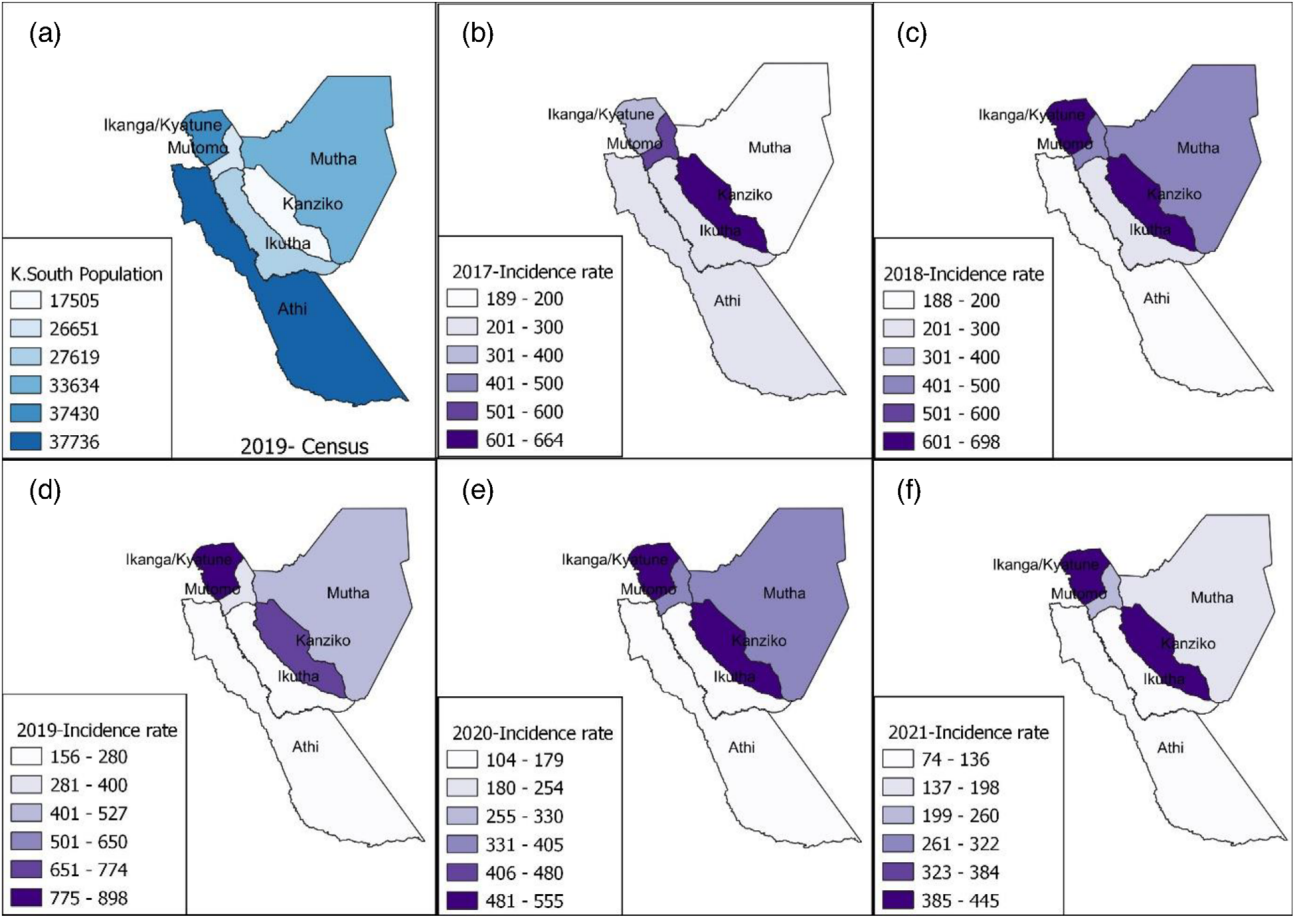
(a–f) Choropleth maps of the population distribution and annual dog‐bite incidence risks per 100,000 persons at the ward level in Kitui South subcounty over the 5‐year period between 2017 and 2021 with the ward‐level population estimates based on the 2019 census and Kenya health information system electronic health system record data.

The spatial distribution of dog‐bite incidence risks at the ward level in Kitui South subcounty per year was also calculated and represented as choropleth maps in Figure 4. The ward populations were based on the 2019 Census data. The dog‐bite incidence risks over the 5‐year period were consistently highest in Kanziko, followed by Ikanga/Kyatune wards.

### Characteristics of dog‐bite cases

The Ikutha Level 4 Hospital reported 212 dog‐bite cases for an estimated 47,500 patients attending during the 1‐year period between 1 October 2018 and 30 September 2019. MMH had a total of 175 dog‐bite cases over the 5‐year period for 71,869 patients attending the hospital between 2017 and 2021. The dog‐bite incidence rate was estimated at 446.3 (407.5–488.3) per 100,000 patients per year at Ikutha Level 4 Hospital and 244.1 (216.2–275.6) per 100,000 patients per year at MMH.

Information on the nature of bites was only reported at the Ikutha Level 4 Hospital and the findings are reported in Table 1. Of the 212 identified dog‐bite cases, 51% (*N* = 108) were aged below 15 years, 53% (*N* = 112) were male victims and 36% (*N* = 77) of the dog‐bite victims were bitten on the limbs, with 25% (*N* = 53) on the legs and 11% (*N* = 24) on the hands. Only 38% (*N* = 81) of the dog‐bite victims received the first anti‐rabies vaccination dose and only 20% (*N* = 43) of the bite victims completed all the five doses. The non‐compliance for treatment by the dog‐bite victims was attributed to lack of funds, forgetfulness on follow‐up treatment, PEP unavailability at the health facility, biting dog being still alive and healing of wounds, among others.^17^ Forty‐four percent (*N* = 93) of the dog‐bite cases took place during the short rainy season (October–December). Only 33% (*N* = 70) of the dogs responsible for the bites were reported as having an owner, while 7.5% (*N* = 16) of the dogs were implicated in more than one dog‐bite event.

**TABLE 1:** Descriptive statistics of dog bites from Ikutha Hospital, Kitui South subcounty, Kenya, between 1 October 2018 and 30 September 2019.

Characteristic	*N* = 212
Age groups (years)	
0–15	108 (51%)
16–35	53 (25%)
36–55	27 (13%)
55+	21 (10%)
Unknown	3 (1%)
Sex	
Female	100 (47%)
Male	112 (53%)
Body part bitten	
Back	2 (0.9%)
Hand	24 (11%)
Leg	53 (25%)
Head	3 (1.4%)
Trunk	3 (1.4%)
Unknown	127 (60%)
Anti‐rabies vaccine doses	
First	81 (38%)
Second	76 (36%)
Third	73 (34%)
Fourth	61 (29%)
Fifth	43 (20%)
Unknown	125 (59%)
Season	
Dry and cool (June–September)	48 (23%)
Dry and hot (January–February)	10 (4.7%)
Long rains (March–May)	61 (29%)
Short rains (October–December)	93 (44%)
Dog ownership status	
Domesticated	70 (33%)
Stray	14 (6.7%)
Wild	1 (0.5%)
Unknown	127 (60%)
Other known bites^a^	
No	43 (20%)
Yes	16 (7.5%)
Unknown	153 (72%)

^a^ These are dogs known to have a history of previous bites.

### Risk factors for dog bites

In the case–control study, the bite cases reported were compared to the controls randomly selected from non‐bite patients in the two hospital registers, with 212 (55%) controls selected from Ikutha Level 4 Hospital and 175 (45%) from the MMH. Among the dog‐bite victims, 54% (*N* = 207) were younger than 15 years and 56% (*N* = 215) were males. In addition, 39% (*N* = 152) of the bite patients visited the hospital during the short rainy season (October–December). Of the control patients presenting to the hospitals, the median age was 32 years (range 1 month–90 years) and 51% (*N* = 198) were male. Fifty‐five percent of the control patients (*N* = 107) visited the hospitals during the dry and cool season (June–September). Twenty‐nine percent (*N* = 114) of the control patients suffered from respiratory tract infections, bronchitis, pneumonia, asthma and dyspnoea. Another 13% (*N* = 51) of the patients had gastrointestinal presentations, which included amoebiasis, diarrhoea, gastroenteritis, gastritis and hyperacidity.

Univariable analysis and descriptive summary results are reported in Table 2. Based on a screening level of *p*‐value less than 0.25, five exposure variables, ward, sex, age group, season and month, were taken forward for evaluation in the multivariable analysis. The hospital variable was retained in the multivariable analysis to account for the stratified sampling by hospital.

**TABLE 2:** Univariable and descriptive analysis for cases and controls for dog‐bite injuries at two hospitals, within Kitui South subcounty, Kenya.

Exposure	Categories	Case (%), *N* = 387	Control (%), *N* = 387	Odds ratio	95% confidence interval	*p*‐Value
Hospital	Ikutha Level 4 Hospital	212 (55%)	212 (55%)	Base	–	>0.99
	Mutomo Mission Hospital	175 (45%)	175 (45%)	1	0.75, 1.33	
Sex	Female	172 (44%)	189 (49%)	Base	–	0.220
	Male	215 (56%)	198 (51%)	1.19	0.90, 1.58	
Ward	Athi	31 (8.0%)	36(9.3%)	Base	–	0.062
	Ikanga	10 (2.6%)	11 (2.8%)	1.06	0.39, 2.83	
	Ikutha	126 (33%)	149 (39%)	0.98	0.58, 1.68	
	Kanziko	10 (2.6%)	8 (2.1%)	1.45	0.51, 4.24	
	Mutha	49 (13%)	30 (7.8%)	1.90	0.98, 3.70	
	Mutomo	153 (40%)	138 (36%)	1.29	0.76, 2.20	
	Other	5 (1.3%)	14 (3.6%)	0.41	0.12, 1.22	
	Unknown	3 (0.8%)	29 (7.8%)	3.48	0.42, 72.4	
Age groups (years)	0–15	207 (54%)	83 (22%)	Base	–	<0.001
	16–35	95 (25%)	115 (31%)	0.33	0.23, 0.48	
	36–55	50 (13%)	75 (20%)	0.27	0.17, 0.41	
	55+	31 (8.4%)	100 (26.8%)	0.12	0.01, 0.53	
	Unknown	3	4	–	–	
Season	Short rains (October–December)	152 (39%)	110 (28%)	Base		<0.001
	Dry and hot (January–February)	29 (8%)	68 (18%)	0.31	0.16, 0.60	
	Long rains (March–May)	86 (22%)	102 (26%)	0.61	0.51, 1.11	
	Dry and cool (June–September)	120 (31%)	107 (28%)	0.81	0.54, 1.22	
Month	January	19 (4.9%)	38 (9.8%)	Base	–	<0.001
	February	10 (2.6%)	30 (7.8%)	0.67	0.26, 1.62	
	March	29 (7.5%)	40 (10%)	1.45	0.70, 3.04	
	April	26 (6.7%)	25 (6.5%)	2.08	0.96, 4.58	
	May	31 (8.0%)	37 (9.6%)	1.68	0.81, 3.51	
	June	29 (7.5%)	28 (7.2%)	2.07	0.98, 4.47	
	July	18 (4.7%)	36 (9.3%)	1.00	0.45, 2.21	
	August	37 (9.6%)	29 (7.5%)	2.55	1.24, 5.39	
	September	36 (9.3%)	14 (3.6%)	5.14	2.29, 12.1	
	October	59 (15%)	41 (11%)	2.88	1.47, 5.77	
	November	50 (13%)	38 (9.8%)	2.63	1.33, 5.34	
	December	43 (11%)	31 (8.0%)	2.77	1.37, 5.78	

Of the variables identified in the univariable analysis, age group and season were significantly associated with the likelihood of sustaining a dog‐bite injury, as illustrated in Table 3. Age was statistically significant; using the age category 0–15 years as the reference group, reduced odds of dog bites with increasing age were observed. For the season variable, with the reference group as the short rainy season, it was observed that dry and hot season was statistically significantly at reduced odds with an odds ratio of 0.31.

**TABLE 3:** Multivariable logistic regression analysis for cases and controls for dog‐bite injuries at two hospitals within Kitui South subcounty, Kenya.

Variable	Categories	Adjusted odd ratio	Confidence interval	*p*‐Value
Age groups (years)	0–15	Base		<0.001
	16–35	0.34	0.24, 0.50	
	36–55	0.26	0.17, 0.41	
	55+	0.13	0.08, 0.21	
Season	Short rains (October–December)	Base		<0.001
	Dry and hot (January–February)	0.31	0.18, 0.53	
	Long rains (March–May)	0.67	0.44, 1.01	
	Dry and cool (June–September)	0.80	0.54, 1.18	
Hospital	Ikutha Level 4 Hospital	Base		0.395
	Mutomo Mission Hospital	1.15	0.84, 1.57	

## DISCUSSION

The study found an annual incidence rate for dog bites between 2017 and 2021 ranging between 234.8 (206.8–264.9) and 433.6 (395.1–474.7) per 100,000 population based on cases reported to the 56 health facilities in Kitui South subcounty. Studies conducted in India and the UK estimated dog‐bite incidence rates of 1700–1960 and 1870 (1100–3180) per 100,000 population per year, respectively.^11^, ^18^ Both studies were cross‐sectional studies in their respective communities with participation of 500 households in Delhi, India, and 385 households in a semi‐rural town in West Cheshire, UK. Furthermore, it is worth noting the lack of dog‐bite studies in Africa, thus limiting the generalisation of the findings. Compared to these studies, the study relied on retrospective data from a health information system and therefore excluded dog‐bite cases that did not require medical attention at the study hospitals. This would suggest that the actual dog‐bite incidence would be greater; however, the current study provides an estimate of dog‐bite injuries of sufficient severity necessitating hospital treatment and is comparable to previously reported estimates in Kenya.

The dog‐bite incidence rate by year had an upward trend from 2017 to 2019, followed by a sudden decline in 2020 and 2021, the latter was probably due to the COVID‐19 pandemic restricting human movement and hospital visits.^19^ Some studies have reported an increase in dog‐bite incidence over time, possibly due to stray dog population growth, which is consistent with our findings prior to the COVID‐19 pandemic.^20^, ^21^ The increasing trend of dog‐bite incidence highlights the inadequacies of the dog control policies (under the rabies control act) in Kenya.^22^ The study findings can contribute towards strengthening of the dog control policies, such as dog population management by advocating for responsible dog ownership. Therefore, the current work would need to continue for more years to evaluate the trends post‐COVID‐19 pandemic.

The data from the 56 health facilities in Kitui South subcounty, conducted over the 5‐year period, revealed that Ikanga/Kyatune and Kanziko wards generally had more dog‐bite cases as a proportion of their estimated population.^15^ Further investigations are needed to identify underlying factors that contribute to the variation in dog‐bite cases in different wards. Additionally, subcounty monitoring would be merited and this information could be used to develop targeted interventions and tailored strategies, such as increased efforts to raise awareness of responsible dog ownership and the availability of rabies vaccines in high burdened locations. These interventions and strategies would reduce dog‐bite incidence and improve access to healthcare services in Kitui county.^23^

Ikutha Level 4 Hospital is a government health facility, whereas MMH is supported by the Catholic church. The distribution of the cases and controls from the two hospitals represented wide coverage of the subcounty, serving six wards from the study subcounty and bordering regions. The dog‐bite incidence rate, based on hospital visiting denominators, was estimated as 446.3 (407.5–488.3) per 100,000 patients per year at Ikutha Level 4 Hospital and 244.1 (216.2–275.6) per 100,000 patients per year at MMH. The difference in the number of dog‐bite cases reported between Ikutha Hospital and MMH could be associated with the accessibility to the hospitals, availability of anti‐rabies vaccines, awareness of reporting dog bites, and size and demographics of the catchment population. Both estimates were, nonetheless, consistent with the overall subcounty wide health system record (KHIS) estimates.

The study found that the majority (36%) of dog‐bite patients were bitten on the limbs (hands and legs), which can be explained by the proximity of the dog to the limbs, especially the legs; moreover, the limbs tend to be used as defence during an attack. This finding is in concurrence with a study conducted in Ogoja‐Nigeria, where the majority of the bites were on the legs and feet as victims protected themselves against the aggressive dogs.^13^, ^21^ Studies in Kenya and Nigeria reported that domesticated (owned) dogs were accountable for more than 80% of the reported dog bites; however, in the current study, only 33% (*N* = 70) of dogs implicated in the bites were known to have owners, with 60% (*N* = 127) of dogs of unknown ownership status, making it difficult to compare with previous studies.^24^, ^25^ Further work is required to assess the role of ownership and free roaming on the risk of bites in the current setting. Nonetheless, the promotion of responsible dog ownership, including improved socialising and reduced free‐roaming dogs, could help contribute to decreased dog‐bite cases in Kitui county.

This retrospective case–control study identified that children under the age of 15 years were at higher risk of being bitten by a dog compared to all other age categories. Studies show that children are more likely to engage in behaviours that provoke aggression from a dog and, due to their smaller stature, may be less threatening to dogs.^26^, ^27^ In addition, studies conducted in Nigeria, Tanzania and Kenya reported that children had a higher risk of dog bite because they are less likely to recognise when a dog is agitated or frightened and thus may not respond appropriately.^21^, ^24^, ^28^ Therefore, it can be hypothesised that the non‐threatening stature of a child and lack of recognition of an aggressive dog increased the risk of dog bites in children. These findings emphasise the value of targeting education and awareness in schools to reduce injuries related to interactions with dogs.

The study found that dog‐bite incidence was lower during the dry and hot season; conversely, the incidence was higher during the short rainy season (October–December). In line with this study, a study in Nigeria reported an increase in dog‐bite cases during the dog breeding season, which corresponds to the short rainy season (October–March).^21^ This could be due to dogs being more aggressive during this period.^29^ However, a 10‐year retrospective study in Nigeria found that dog bites were more prevalent during the dry season (summer). They attributed this to an increase in human recreational activities and contact between dogs and humans.^30^ The differences in the findings of the two studies may at least in part be attributed to the difference in the population density in the two settings, with about 615 individuals per square kilometre in Enugu, an urban region in southeast Nigeria, while Kitui is a rural region in Kenya, with a density of 37 people per square kilometre.^31^, ^32^ In summary, a number of factors may contribute to increased risk, but in Kitui South, the short rainy season would appear to be an important period to be particularly vigilant.

This study also observed that over 50% of the dog‐bite victims were males; however, sex was not a significant factor in the current study. In contrast, previous studies conducted in Bhutan, South Africa and Kenya reported that males were significantly more likely to be bitten by dogs than females.^13^, ^33^, ^34^ The lack of sex as a significant factor can possibly be attributed to the limited power of the study; it warrants further investigation to confirm the association between the sex of an individual and the occurrence of dog bites.

There were several limitations in the study, including the fragmented nature of the health recording system, which hindered acquiring complete data on dog bites and their management. Ikutha Level 4 Hospital had a paper‐based recording system whereas electronic records were obtained from MMH. The retrospective nature of this study limited control of the variables recorded and had potential for missing data. Further prospective studies to build on this retrospective study are recommended to address such limitations.

Additionally, in the case–control study, the datasets came from overlapping but different time periods, with a 5‐year study period from MMH between January 2017 and December 2021 and a 1‐year study period from Ikutha Level 4 Hospital between October 2018 and September 2019. The difference was attributed to the availability and access of the paper‐based data specifically from Ikutha Level 4 Hospital. The use of the stratification of control selection and adjusting for this in the analysis was undertaken to adjust for such sampling. Finally, ideally, other dog factors (age, breed, sex and dog rabies vaccination status) would have been evaluated in the risk factor analysis; however, these data were not recorded in the hospital databases; therefore, further work is merited to explore animal‐related risk factors for bite injuries.

In summary, this study described the frequency and distribution of dog bites and identified major risk factors for dog bites in Kitui South subcounty. Children under 15 years of age and those in the short wet season were identified as having increased odds of dog‐bite occurrence. This information could help utilise limited resources for greater impact by targeting specific high‐risk groups and seasons in Kitui county for education and prevention. The findings support the need for promotion of responsible dog ownership, enhanced access to PEP resources in high dog‐bite burdened regions, education on the risks of rabies among school‐going children and regular mass dog vaccination campaigns with consideration of the seasonality in Kitui South subcounty. Our study endorses evidence‐based decision making that will reinforce dog control policies in Kenya, thus contributing to the elimination of dog‐mediated rabies by 2030.^35^

## AUTHOR CONTRIBUTIONS

The study was conceptualised by Peris Kung'u, with the study design being refined by David Brodbelt and Peris Kung'u. Data collection and analysis were conducted by Peris Kung'u guided by David Brodbelt. Peris Kung'u developed the initial draft manuscript, which was revised by David Brodbelt. David Brodbelt and Peris Kung'u reviewed the final manuscript before submission.

## CONFLICTS OF INTEREST STATEMENT

The authors declare they have no conflicts of interest.

## ETHICS STATEMENT

The study was granted ethical approval by the Ethical Review Committee at the Royal Veterinary College (URN number: SR 2022‐0109).

## Data Availability

The summary data are available from the authors on request.
